# Genetic Analysis and QTL Mapping of Fruit Peduncle Length in Cucumber (*Cucumis sativus* L.)

**DOI:** 10.1371/journal.pone.0167845

**Published:** 2016-12-09

**Authors:** Zi-Chao Song, Han Miao, Song Zhang, Ye Wang, Sheng-Ping Zhang, Xing-Fang Gu

**Affiliations:** Institute of Vegetables and Flowers, Chinese Academy of Agricultural Sciences, Beijing, China; United States Department of Agriculture, UNITED STATES

## Abstract

Mechanized harvesting of cucumbers offers significant advantages compared to manual labor as both shortages and costs of labor increase. However the efficient use of machines depends on breeding plants with longer peduncles, but the genetic and molecular basis of fruit peduncle development in cucumber is not well understood. In this study, F_2_ populations were developed from a cross between two inbred lines, 1101 with a long peduncle and 1694 with a short peduncle. These were grown at two field sites, Hainan, with a tropical marine climate, in December 2014, and Beijing, with a warm temperate climate, in May 2015. Electron microscope examination of the pith cells in the peduncles of the two parental lines showed that line 1101 had significantly greater numbers of smaller cells compared to line 1694. The inheritance of cucumber fruit peduncle length (FPL) was investigated by the mixed major gene and polygene inheritance model. Genetic analysis indicated that FPL in cucumber is quantitatively inherited and controlled by one additive major gene and additive-dominant polygenes (D-2 model). A total of 1460 pairs of SSR (simple sequence repeat) primers were analyzed to identify quantitative trait loci (QTLs). Two similar genetic maps with 78 SSR markers which covered 720.6 cM in seven linkage groups were constructed based on two F_2_ populations. QTL analysis from the data collected at the two field sites showed that there are two minor QTLs on chromosome 1, named *qfpl1*.*1* and *qfpl1*.*2*, and one major QTL on chromosome 6, named *qfpl6*.*1*. The marker UW021226, which was the closest one to *qfpl6*.*1*, had an accuracy rate of 79.0% when tested against plants selected from populations of the two parents. The results from this study provide insights into the inheritance and molecular mechanism of the variation of FPL in cucumber, and further research will be carried out to fine map *qfpl6*.*1* to develop more accurate markers for MAS breeding.

## Introduction

Cucumber, *Cucumis sativus* L., is one of the most important cultivated vegetable crops, ranking 4^th^ in quantity of world vegetable production. Three fourths of this is produced in China where both the area harvested and the quantity produced increase year by year [1]. However, the cultivation and harvesting of cucumbers is very labor intensive and the increase in planting area is being impeded by a shortage of labor and rising employment costs. Consequently, mechanization of harvesting is becoming more and more important in the process of cucumber production. Mechanical harvesters have been used for a range of crops, including cucumber [2,3], orange [4], strawberry [5], tomato [6], eggplant [7,8], sweet-pepper [9] and apple [10]. The key to successful operation of mechanical harvesters is the extent to which the machine can distinguish between fruit, peduncle, leaf, and stem, and then grip the fruit and cut the peduncle correctly, and the longer the peduncle the more successful will be this process.

Many different traits relating to the quality of cucumber fruit have been reported, including the length, weight, and diameter of fruit [11–13], uniform immature fruit color [14,15], glossy fruit skin [15,16], warty fruit [17], and yellow fruit flesh[18], but so far, peduncle length in cucumber has received little attention.

Cui et al. [19] reported on the inheritance of fruit peduncle length in luffa using the mixed major gene and polygene inheritance model developed by Gai et al. [20]. They found that the fruit peduncle length of luffa is controlled by one additive-dominant major gene and several additive-dominant-epistasis polygenes (D-0 model), and that the major gene showed heritabilities of 57.60% and 61.90% in the F_2_ and BC_1_P_1_ generations respectively. Chen et al. [21] found that the fruit peduncle length in summer squash is controlled by two additive-dominant-epistasis major genes (B-1 model), and Chen at al. [22] found that, in pepper, two complete dominant major genes and several additive-dominant polygenes controlled fruit peduncle length. However, so far, there are no reports on the heritability of peduncle length of cucumber.

At the molecular level, Yuan et al. [11,12] identified several quantitative trait loci (QTLs) related to peduncle length of cucumber in an F_2_ population, and also produced F3 families and a RIL population from the same cross. Among these QTLs, *fpl2*.*1*, *fpl2*.*2*, and *fpl6*.*1* were detected in different populations and seasons respectively, but they accounted for only a low amount of the variability between phenotypes (from 3.17% to 8.87%) and have not been mapped to any of the chromosomes.

In the present study, two inbred lines, one with a long fruit peduncle and the other with a short fruit peduncle, were crossed to construct genetic populations for inheritance analysis and chromosomal mapping of the related QTLs. The results from this study will promote the breeding of new cultivars of cucumbers that would be better adapted to mechanical harvesting and thus obviate the problems of both shortage of labor and higher costs.

## Materials and Method

### Plant materials

Inbred line 1101 with long fruit peduncles and inbred line 1694 with short fruit peduncles were used as parental lines to develop a segregating population for inheritance analysis and QTL mapping. 1101, the female parent (P_1_), is from northern Europe with an average fruit peduncle length (FPL) of more than 5.5 cm whereas 1694, the pollen donor (P_2_), is from southern China with an average FPL of less than or equal to 2 cm. The F_1_ population was self-pollinated to generate the F_2_ population, and the F_1_ population was backcrossed with either 1101 to generate BC_1_P_1_ or with 1694 to generate BC_1_P_2_.

All plants were grown at two sites: the Sanya Science and Technology Academy for Crop Winter Multiplication at Sanya, Hainan in December, 2014 (18°15' N, 109°30' E; average day/night temperatures 22°C /19°C; daylength ~11 hr) and in the greenhouse of the Institute of Vegetables and Flowers, Chinese Academy of Agricultural Science at Shunyi, Beijing in May, 2015 (40°10' N, 116°51' E; average day/night temperatures 26°C/14°C; daylength ~14 hr). Nineteen cucumber lines, including twelve with FPL > 5.0cm and seven with FPL < 1.2cm, were used to validate the molecular markers closest to the major QTL.

### Histology

Fruit peduncles were collected from lines 1101 and 1694 14 days after anthesis. Samples for paraffin embedding were fixed in FAA (3.7% formaldehyde, 5% glacial acetic acid and 50% ethanol) and stored at room temperature for further embedding. The fixed samples were dehydrated in a graded series of ethanol (70%, 85%, 95%, and 100%), followed by a xylene/ethanol series (xylene:ethanol 1:3, 1:1, and 3:1) and finally 100% xylene. Xylene was replaced gradually with paraffin (Paraplast Plus, Sigma, P3683) at 60°C for two nights with four times replacement of paraffin. Ten μm sections were made using a HEISTION ERM3000 microtome and stained with Toluidine Blue O [23], and examined at x100 in a light microscope.

### Phenotype measurement and statistics

The peduncle lengths of three to five fruits of each plant were measured from the peduncle bottom to the joint with the stem with a precision of 0.1 cm when the fruits were commercially mature. The data were analyzed using Microsoft Excel 2013 using the mixed major gene and polygene inheritance model of Gai et al. [20,24].

### Quantitative trait inheritance analysis

The continuous phenotypic distribution of peduncle length was analyzed using the maximum likelihood estimation via the IECM algorithm [25]. According to the Akaike Information Criterion (AIC) values, a few inheritance models were selected as alternative models, and then fitness tests, including the uniformity test and the Kolmogorov-Smirnov test, were applied to find the significantly different statistics of each alternative model. Finally, the model with the least significantly different statistics was selected as the optimum inheritance model. Based on this optimum inheritance model, the first order parameters (genetic effects of major genes and polygenes) and second order parameters (heritability values of major genes and polygenes) were estimated by the method of least squares. The heritability values of major genes ($h_{mg}^{2}$) and polygenes ($h_{pg}^{2}$) were expressed as follows:

$$h_{mg}^{2}\;=\;\sigma_{mg}^{2}÷\sigma_{p}^{2}$$

$$h_{pg}^{2}\;=\;\sigma_{pg}^{2}÷\sigma_{p}^{2}$$

($\sigma_{mg}^{2}$: the variance of major gene, $\sigma_{pg}^{2}$: the variance of polygenes, $\sigma_{p}^{2}$: the phenotypic variance)

### DNA extraction and SSR marker analysis

Genomic DNA was extracted from young leaf tissue of the parents P_1_ and P_2_, and the F_1_ and F_2_ populations using a modified CTAB extraction procedure [26]. The concentration and quality was determined after electrophoresis on 1% (w/v) agarose gels and then diluted with distilled water to 15 ng/uL. 1,288 pairs of SSR markers were selected from the genetic map of the cucumber genome produced by Ren et al. [27] and 122 pairs from the study of Cavagnaro et al. [28]. A further 50 pairs were designed with Primer 5.0 software (http://www.PremierBiosoft.com) based on the genome sequence of 'Chinese long' inbred line 9930 [29] at the preliminary mapping region of the major QTL. All primers were screened on the two parents, and polymorphic primers were applied to the F_2_ population for linkage construction and QTL analysis.

PCR amplification was carried out in a volume of 10 uL, containing 3 uL of DNA (15 ng/uL), 1 uL of both forward and reverse primers (50 ng/uL), and 5 uL of Go Taq Green Master Mix (Promega, USA). The PCR program was as follows: denaturation at 94°C for 4 min, 35 cycles of denaturation at 94°C for 15 s, annealing at 55°C for 15 s, and extension at 72°C for 30 s, with a final extension at 72°C for 5 min. Amplified products were separated on 6.0% non-denaturing polyacrylamide gels at 150 V for 1 h, and the bands were visualized and photographed after silver staining.

### Linkage map construction and QTL mapping

JoinMap 4.0 software [30] was used to generate the linkage map. Segregation distortion at each marker locus was tested against the expected 1:2:1 or 3:1 ratios for the F_2_ population using the Chi-squared test. Linkage groups were determined with a minimum logarithm of odds (LOD) likelihood score of 3.0 and a recombination fraction of 0.3. Genetic distances between markers were calculated with the Kosambi mapping function [31]. An interval mapping analysis [32] was conducted by using MapQTL4.0 [33] to detect QTLs. Permutation tests were conducted to assess the LOD threshold at the α = 0.05 level. The possibility of QTL existence was scanned on every chromosome at intervals of 1 cM. QTLs that were detected were verified by the Multiple-QTL model (MQM) and markers at the position of the highest LOD score were selected as cofactors. Each locus was named by an abbreviation of the trait followed by the chromosome (Chr.) number and locus number [13,34].

## Results

### Comparison of cell morphology in the parental lines

**Fig 1:**
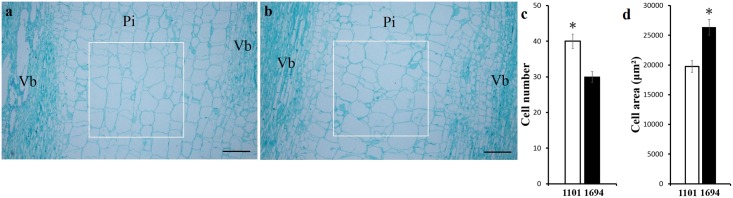
Comparison of cell morphology in the parental lines 1101 and 1694. a-b Microscopic longitudinal sections of the fruit peduncle 14 days after anthesis in line 1101 (a) and line 1694 (b) (Vb, vascular bundles; Pi, pith). The dimensions of the white outlined box is 889 μm × 889 μm, and the black bar is 250 μm. c-d The cell number (c) and cell size (d) were approximately calculated. The bars show significance calculated by the unpaired t test, P<0.05.

Longitudinal sections of peduncles in the parental lines showed that both the number and size of the cells differed in the pith tissue between the vascular bundles. There were significantly more cells per unit area in line 1101 compared to line 1694, and the cells were significantly smaller (Fig 1)

### Inheritance of fruit peduncle length in cucumber

**Fig 2:**
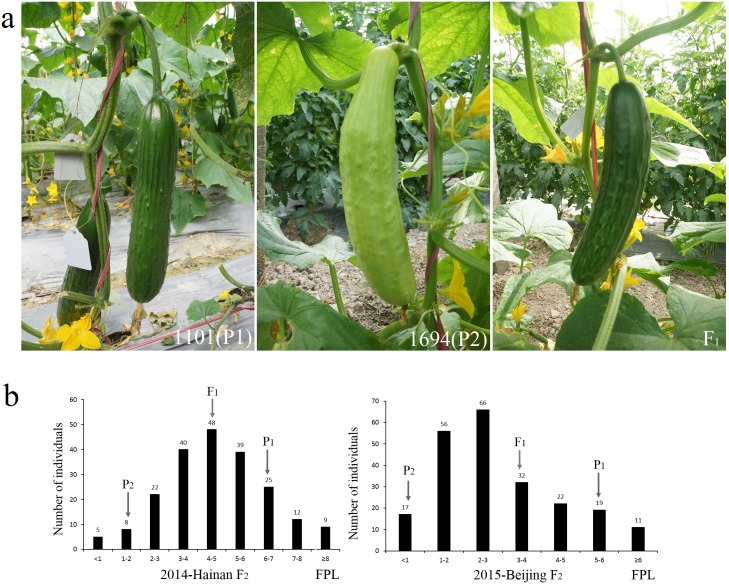
Cucumber fruit peduncle length (FPL) performance of two parents and their F1 progeny, and frequency distribution of FPL among different populations at Hainan in December 2014 and Beijing in May 2015. a 1101 (P1, left), 1694 (P2, middle) and their F1 (right). 1101 had longer fruit peduncles than 1694, and F1 between them. b The frequency of fruit peduncle length of P1, P2, F1 and F2 populations at Hainan in December, 2014 and Beijing in May, 2015.

Fruit peduncle lengths for line 1101 (P_1_) at Hainan in 2014 and at Beijing in 2015 varied from 4.50 to 7.50 cm and 5.10 to 6.70 cm, respectively whereas for line 1694 (P_2_) at the two sites the lengths varied from 0.90 to 3.50 cm and 0.50 to 1.50 cm, respectively. The mean fruit peduncle lengths of F_1_ at Hainan and Beijing were 4.60 cm and 2.90 cm, respectively (S1 Table). The frequency distributions of the fruit peduncle length in the F_2_ populations at both sites were both normal and skew normal (Fig 2b) suggesting that fruit peduncle length in cucumber is quantitatively inherited.

Values calculated for both AIC and the maximum likelihood function for the mixed major gene and polygene model suggested 23 kinds of inheritance (S2 Table). These included 4 A models (controlled by one major gene), 6 B models (controlled by two major genes), 2 C models (controlled by polygenes), 5 D models (controlled by one major gene and several polygenes), and 6 E models (controlled by two major genes and polygenes). According to the minimum AIC value, the D-1, D-2, and D-4 models were selected as the candidates, and after applying tests for goodness of fit (S3 Table), D-2 was selected as the optimum inheritance model indicating that cucumber fruit peduncle length is controlled by one additive major gene and additive-dominant polygenes.

Estimates of the first order parameters for the D-2 model indicated that the additive gene effect was higher than the dominant effect, and that parental line 1101 with long fruit peduncles contributed to the latter. However there were differences in heritability between the two sites. For example, the heritability of the major gene in the BC_1_P_1_ generation at Hainan was significantly higher than that for the polygenes, 53.30% compared to 5.80%, whereas it was slightly lower than polygenes at Beijing, 42.60% compared to 49.40%. On the other hand, the heritability of the major gene in BC_1_P_2_ and F_2_ generations was lower than that for the polygenes at Hainan but it was the opposite at Beijing (Table 1). The environmental variance accounts for 20.2% to 80.0% of the phenotypic variance at Hainan, and 7.3% to 66.4% at Beijing respectively.

**Table 1:** Estimates of genetic parameters of the D-2 model at Hainan and Beijing.

Time	1^st^ order parameter	Estimation	2^nd^ order parameter	Estimation		
				B_1_	B_2_	F_2_
	m	3.97	σ_*p*_^2^	1.53	0.78	3.09
	d	1.04	σ_*mg*_^2^	0.81	0.02	0.87
2014-Hainan	[d]	1.11	σ_pg_^2^	0.09	0.14	1.6
	[h]	0.51	σ^2^	0.63	0.63	0.63
			h_*mg*_^2^ (%)	53.30%	2.10%	28.10%
			h_*pg*_^2^ (%)	5.80%	17.80%	51.70%
	m	3.26	σ_*p*_^2^	3.12	0.34	2.56
	d	1.49	σ_*mg*_^2^	1.33	0.09	1.61
2015-Beijing	[d]	0.98	σ_pg_^2^	1.54	0	0.69
	[h]	-0.6	σ^2^	0.25	0.25	0.25
			h_*mg*_^2^ (%)	42.60%	26.60%	63.10%
			h_*pg*_^2^ (%)	49.40%	0.00%	27.10%

m, the average of population; d, additive effect of major gene; [d], additive effect of polygene; [h], dominant effect of polygene; σ_*p*_^2^, phenotypic variance; σ_*mg*_^2^, variance of major gene; σ_pg_^2^, variance of polygene; σ^2^, the environmental variance; h_*mg*_^2^ (%), major gene heritability; h_*pg*_^2^ (%), polygene heritability.

### Linkage map construction

**Fig 3:**
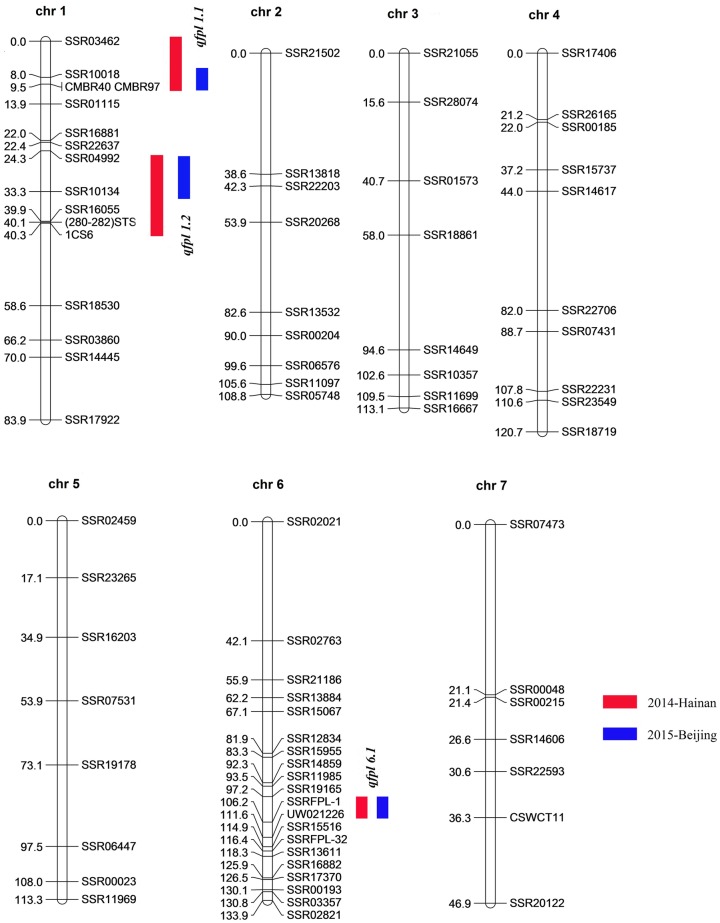
Genetic map of cucumber with the locations of putative QTLs for fruit peduncle length based on F_2_ populations at Hainan in 2014. Three QTLs were detected at the same location at both Hainan and Beijing, respectively. *qfpl1*.*1* and *qfpl1*.*2* were identified on chromosome 1 (chr.1), and *qfpl6*.*1* was detected on chr.6. Map distance is given in centimorgans (cM). Note: Red symbol indicates Hainan in 2014, Blue symbol indicates Beijing in 2015.

After screening a total of 1,460 SSR markers, 236 (16.2%) were selected according to polymorphisms between the two parents. 78 markers scattered on cucumber chromosomes with proportional spacing and these were used to construct the linkage map (S4 Table). Based on the F_2_ populations at the two sites, two genetic maps containing seven linkage groups were constructed. Because the populations at Hainan and Beijing were of different sizes, 225 and 235 individuals respectively, the genetic distances between the markers were different, but the order of markers in each linkage group was coincident (S1 Fig). The genetic map for the F_2_ population at Hainan is graphically presented in Fig 3, and scans 720.6 cM with an average marker interval of 9.24 cM. The order of the markers was highly consistent with their physical location in the genome of 9930. Of the 78 makers mapped, Chi-square tests indicated that 10 (12.8%) showed segregation distortion with the F_2_ population (S4 Table), four biased toward 1101 and six favored 1694.

### QTL mapping analysis

**Fig 4:**
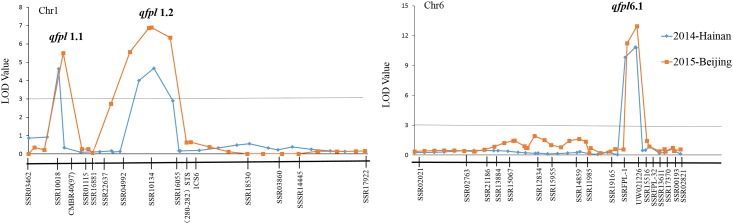
QTL analysis of cucumber fruit peduncle lengths at Hainan and Beijing.

The genetic map was used to detect QTLs for fruit peduncle length. After a first round of interval mapping (IM), markers with the highest LOD value were selected as cofactors for MQM mapping with the MapQTL4.0 software. Coincidently, both IM and MOM mapping approaches detected the same QTLs for the two sites. Two QTLs detected on chromosome 1 were named *qfpl1*.*1* and *qfpl1*.*2*, and another located on chromosome 6 was named *qfpl6*.*1*. LOD scores respectively for the *qfpl1*.*1* and *qfpl1*.*2* were 4.65 and 4.67 at Hainan, and both accounted for 9.80% of the phenotypic variation. In 2015-Beijing, LOD scores respectively for the *qfpl1*.*1* and *qfpl1*.*2* were 5.15 and 6.9, and accounted for 7.70% and 10.50% of the phenotypic variation. And *qfpl6*.*1* on chromosome 6 was located at 111.2 cM between markers SSRFPL-1 and UW021226 with LOD scores of 10.84 (R^2^ = 21.50%) at Hainan, and 12.95 (R^2^ = 27.50%) at Beijing respectively (Fig 4 and Table 2). A permutation test showed that the LOD threshold was 2.5 to obtain 95% confidence of detecting a putative QTL. The additive effects for these three QTLs at the two sites were all positive.

**Table 2:** QTLs controlling the length of cucumber fruit peduncle and their effects.

Seasons	QTL	Chromosome (chr.)	Marker Interval	LOD	R^2^/%	Additive Effects
2014-Hainan	*qfpl1*.*1*	1	SSR03462-CMBR40(97)	4.65	9.80%	0.77
	*qfpl1*.*2*	1	SSR04992-1CS6	4.67	9.80%	0.76
	*qfpl6*.*1*	6	SSRFPL-1-UW021226	10.84	21.50%	1.13
2015-Beijing	*qfpl1*.*1*	1	SSR10018-CMBR40(97)	5.15	7.70%	0.53
	*qfpl1*.*2*	1	SSR04992-SSR10134	6.9	10.50%	0.82
	*qfpl6*.*1*	6	SSRFPL-1-UW021226	12.95	27.50%	1.22

### Validation of molecular markers linked to the *qfpl6*.*1* locus for MAS breeding

Marker UW021226, the closest to *qfpl6*.*1* with a genetic distance of 0.4 cM, was tested on 19 cucumber inbred lines, twelve with FPL greater than 5.0 cm and seven with FPL less than 1.2 cm. Three accessions with long FPL (CG39, CG50, CG106) did not match 1101, and one with short FPL (CG90) did not match 1694, suggesting that the accuracy of this marker in a MAS breeding program would be 79.0% (Table 3).

**Table 3:** Validity of the marker UW021226 tightly linked to *qfpl6*.*1* was tested using 19 accessions of cucumber germplasm.

Material code	Fruit peduncle length/cm	UW021226	Material code	Fruit peduncle length/cm	UW021226
1101	5.10 ~6.70	a	CG45	0.4	b
1694	0.50 ~1.50	b	CG50	5.25	b
F_1_	2.10 ~4.10	h	CG57	5.07	a
CG11	0.95	b	CG90	0.97	a
CG15	1.1	b	CG91	5.67	a
CG26	5.72	a	CG94	5.86	a
CG30	5.41	a	CG99	5.45	a
CG33	5.74	a	CG104	5.74	a
CG37	1.07	b	CG106	6.84	h
CG39	5.38	b	CG111	0.82	b
CG40	5.69	a	CG116	0.85	b

The phenotypic measurement was conducted in May, 2015 at Beijing.

## Discussion

The inheritance of fruit peduncle length (FPL) in cucumber was studied with six generations based on a cross between parents with long, 1101, and short, 1694, peduncles. These were P_1_, P_2_, F_1_, F_2_, BC_1_P_1_, and BC_1_P_2_. Plants were grown at two sites: Hainan in December (18°15' N, 109°30' E; average day/night temperatures 22°C /19°C; daylength ~11 hr) and Beijing in May (40°10' N, 116°51' E; average day/night temperatures 26°C/14°C; daylength ~14 hr). FPL showed a continuous distribution that was found to fit a quantitative inheritance model, although there were differences in average FPL between the two sites in each generation that were attributed to the different environments. This was particularly evident for the BC_1_P_1_ generation which showed an environmental variance as high as 80.1%. Lin et al [35] reported on the vitamin C content in non-heading Chinese cabbage and found that the environmental variance in the BC_1_P_2_ generation varied from 24.50% to 77.02%. In the present study, the heritability of the BC_1_P_1_ and F_2_ generations at the two sites varied from 59.1% to 92.0%.

FPL was found to be controlled by one additive major gene and several additive-dominant polygenes, and agrees with the finding by Cui et al [19] in luffa that fruit peduncle length is mainly controlled by one major gene with several minor polygenes. In addition, there was a stabilized higher major gene heritability ($h_{mg}^{2}$) in the BC_1_P_1_ generation (53.30% and 42.60%) under different environments (Table 1) suggesting that early selection in this generation would be beneficial in a breeding program.

For QTL analysis, two F_2_ populations were constructed from the cross between 1101 and 1694 and planted at Hainan in 2014 and Beijing in 2015. Two minor QTLs, *qfpl1*.*1* and *qfpl1*.*2* were located on chromosome 1. A major QTL, *qfpl6*.*1*, located on chromosome 6, was defined by two flanking SSR markers, SSRFPL-1 and UW021226, with LODs of 10.84 and 10.95 that accounted for 21.50% and 27.50% of the total phenotypic variance respectively (Table 2). Yuan et al. [12] found a QTL for FPL in cucumber, named *fpl2*.*2*, which was located between the *F* gene and SSR marker CSWCT25 in an F_2_ population and its F_2:3_ families. Later, using a RIL population developed from the same parental lines, Yuan et al. [11] detected two QTLs named *fpl2*.*1*, located between the *F* gene and SSR marker CS30, and *fpl6*.*1*, located between the *ss* gene and *D* gene. However, all of these QTLs accounted for a low phenotypic variation (~8.87%). After comparing the QTLs reported in the present study with those of Yuan et al. [11,12], marker CS30, the closest to *fpl2*.*1*, was selected to conduct a blast alignment on the genome of 9930 [29]. The position of the forward primer (23,781,324 bp) was located in the region of *qfpl6*.*1* (22,690,272 to 23,873,950 bp) suggesting the possible existence a major gene controlling FPL in this region. Since most of the flanked markers of the QTLs reported by Yuan et al [11,12] were morphological markers, and were located at relatively large genetic distances from the QTLs, their application in breeding is restricted.

The validation of the link between marker UW021226 and QTL *qfpl6*.*1* was tested using 19 cucumber inbred lines. The accuracy rate of UW021226 for selecting long or short peduncles was 79%, and this low value is attributed to the existence of minor polygenes, and the effect of environment on peduncle length. Compared with the markers linked to qualitative traits [14, 18, 36], 79% is considered to be too low for accurate MAS breeding. Therefore, further research will be aimed at developing a marker that co-segregates with the gene for FPL to increase breeding efficiency.

The major QTL, *qfpl6*.*1*, was delimited to an 1183 kb physical interval on chromosome 6, and 179 genes were predicted in this region [37]. Two adjacent genes *Csa6G492310* and *Csa6G493310* brought our attention, and they belong to Gretchen Hagen 3 (*GH3*) gene families encoding an auxin-responsive promotor with a central function in the auxin signaling transduction pathway [38, 39]. The results of blast alignment in the Uniprot database (http://www.uniprot.org/) showed that their homologous genes in *Arabidopsis thaliana*, with identities of 74.3% and 76.6%, respectively, had similar functions in catalyzing the synthesis of indole-3-acid (IAA)-amino acid conjugates, providing a mechanism for the plant to modify auxin levels [40].

Comparison of the number and size of the cells in the pith of the parental lines shown by longitudinal sections of the peduncle (Fig 1a and 1b)., suggests that there were differences in cell division and cell expansion in 1101 and 1694. Based on these results, we speculated that the predicted genes *Csa6G492310* and *Csa6G493310* probably participated in the regulation of fruit peduncle development in cucumber by mediating the cell division and cell expansion. However, further fine mapping is needed to narrow the mapping region and to validate this result.

This research will lead to the development of a marker for FPL in hybrid cucumber production and facilitate marker-assisted selection (MAS) of the long fruit peduncle trait in cucumber breeding. It will also lead to an understanding of the way in which the fruit peduncle develops and the future fine mapping and cloning of the *qfpl6*.*1*.

## Supporting Information

S1 Fig Genetic map of cucumber based on F_2_ populations at Beijing in 2015. (TIF)

S1 Table Measured value of fruit peduncle length in cucumber and some genetic parameters in F_2_ and backcrossed populations in 2014 and 2015. Letters indicate significant differences among trait values at P = 0.05 level.
(DOCX)

S2 Table Values of maximum likelihood function and values of Akaike`s information criterion (AIC) obtained from IECM algorithm. (DOCX)

S3 Table Tests for goodness of fit of the preliminary selected inheritance model at the two sites. *U*_1_^2^, *U*_2_^2^, *U*_3_^2^ are the statistic of Uniformity test; _*n*_*W* ^2^ is the statistic of Smimov test; *D*_*n*_ is the statistic of Kolmogorov test. The critical of _*n*_*W* ^2^ is 0.461 at 0.05 level. * indicates the different significance at *P* <0.05 level.
(DOCX)

S4 Table Information of 78 SSR markers placed on the 1101×1694 linkage map. Loci with asterisks show segregation distortion.
(DOCX)
