# Supplementary figures and images for: Genetic Analysis and QTL Mapping of Fruit Peduncle Length in Cucumber (Cucumis sativus L.)
Source: PLoS One. 2016 Dec 9;11(12):e0167845. doi: 10.1371/journal.pone.0167845 (PMC5148027; doi:10.1371/journal.pone.0167845)

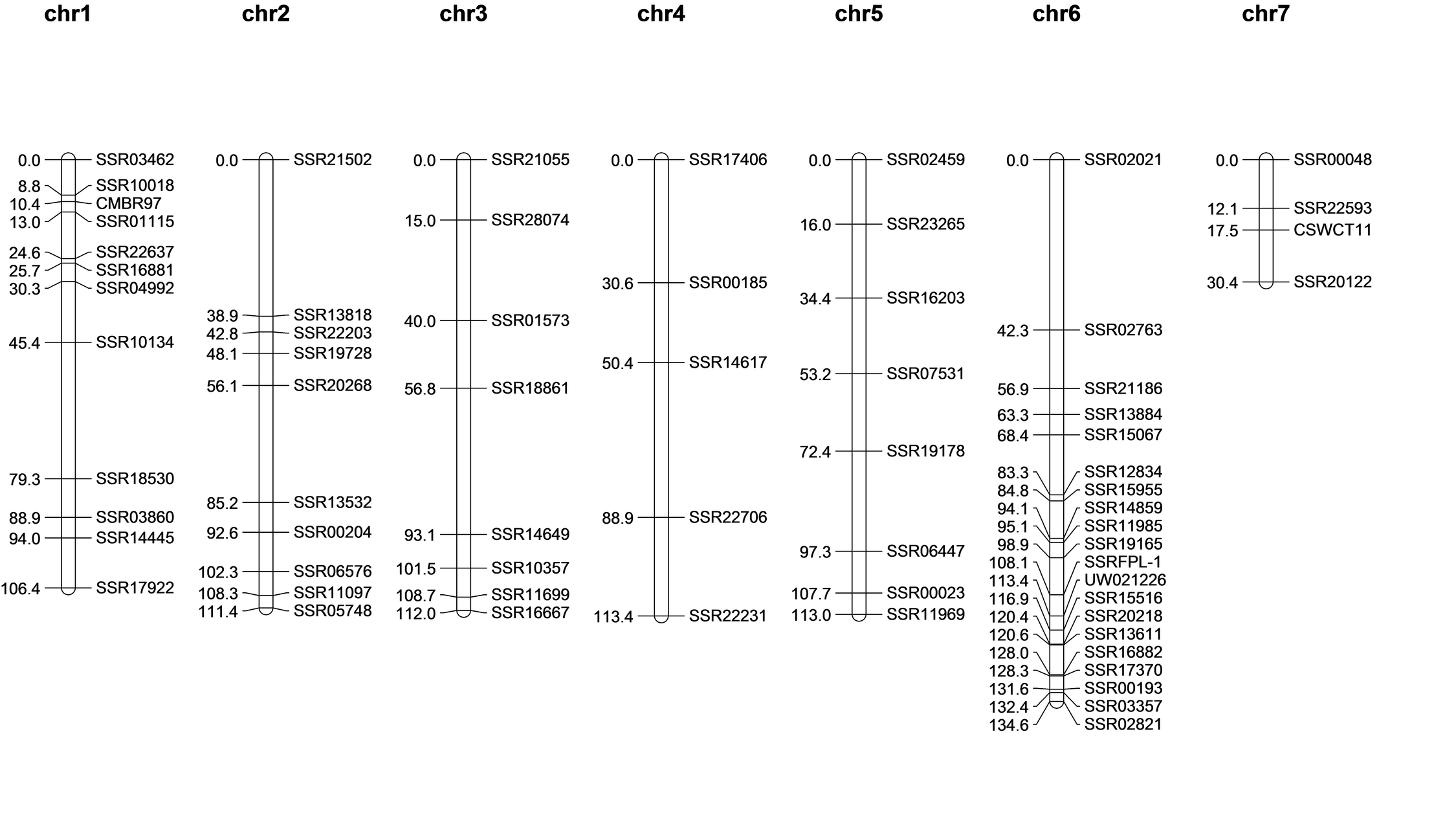

Supplement: S1 Fig — (TIF) [file pone.0167845.s001.tif]
