# Supplementary material for: Genetic Analysis and QTL Mapping of Fruit Peduncle Length in Cucumber (Cucumis sativus L.)
Source: PLoS One. 2016 Dec 9;11(12):e0167845. doi: 10.1371/journal.pone.0167845 (PMC5148027; doi:10.1371/journal.pone.0167845)
Supplement: S1 Table — Letters indicate significant differences among trait values at P = 0.05 level. (DOCX) [file pone.0167845.s002.docx]

**S1 Table. Measured value of fruit peduncle length in cucumber and some genetic parameters in F_2_ and backcrossed populations in 2014 and 2015.**

| Time | Generations | Mean ± S.E. | Variation range | Skewness | Kurtosis |
| --- | --- | --- | --- | --- | --- |
| 2014-Hainan | P_1_ | 6.28±0.42 a | 4.50 ~7.50 | - | - |
|  | P_2_ | 1.89±0.23 c | 0.90 ~3.50 | - | - |
|  | F_1_ | 4.60±0.28 b | 3.50 ~6.00 | - | - |
|  | F_2_ | 4.58±0.12 | 1.00 ~9.25 | 0.21 | -0.13 |
|  | BC_1_P_1_ | 4.91±0.14 | 1.75 ~7.75 | 0.08 | -0.38 |
|  | BC_1_P_2_ | 2.76±0.1 | 1.00 ~6.00 | 0.44 | 1.43 |
| 2015-Beijing | P_1_ | 5.66±0.15 a | 5.10 ~6.70 | - | - |
|  | P_2_ | 1.10±0.17 c | 0.50 ~1.50 | - | - |
|  | F_1_ | 2.91±0.27 b | 2.10 ~4.10 | - | - |
|  | F_2_ | 2.88±0.11 | 0.4 ~10.2 | 1.01 | 1.32 |
|  | BC_1_P_1_ | 3.89±0.19 | 1.00 ~8.30 | 0.44 | -0.16 |
|  | BC_1_P_2_ | 1.25±0.08 | 0.40~2.60 | 0.54 | -0.57 |

Letters indicate significant differences among trait values at P = 0.05 level
