# Supplementary material for: Genetic Analysis and QTL Mapping of Fruit Peduncle Length in Cucumber (Cucumis sativus L.)
Source: PLoS One. 2016 Dec 9;11(12):e0167845. doi: 10.1371/journal.pone.0167845 (PMC5148027; doi:10.1371/journal.pone.0167845)
Supplement: S2 Table — (DOCX) [file pone.0167845.s003.docx]

**S2 Table.** **Values of maximum likelihood function and values of Akaike`s information criterion（AIC）obtained from IECM algorithm.**

| Model | 2014-Hainan | | 2015-Beijing | | Model | 2014-Hainan | | 2015-Beijing | |
| --- | --- | --- | --- | --- | --- | --- | --- | --- | --- |
|  | Maximum  likelihood | AIC | Maximum  likelihood | AIC |  | Maximum  likelihood | AIC | Maximum  likelihood | AIC |
| A-1 | -702.4 | 1412.8 | -672.2 | 1352.4 | D-0 | -672.4 | 1368.9 | -636.7 | 1297.3 |
| A-2 | -703.2 | 1412.3 | -698.5 | 1403 | D-1 | -673.6 | 1365.2 | -635.5 | 1289 |
| A-3 | -720.7 | 1447.3 | -732.7 | 1471.4 | D-2 | -673.6 | 1363.2 | -635.5 | 1287 |
| A-4 | -731.5 | 1469.1 | -686 | 1378 | D-3 | -676.4 | 1368.9 | -654.7 | 1325.4 |
| B-1-1 | -673.4 | 1366.8 | -655.8 | 1331.7 | D-4 | -675.6 | 1367.3 | -635.8 | 1287.6 |
| B-1-2 | -687.7 | 1387.4 | -659 | 1330 | E-1-0 | -669 | 1374 | -632.9 | 1301.7 |
| B-1-3 | -732.5 | 1472.9 | -722.6 | 1453.1 | E-1-1 | -672.5 | 1375 | -632.8 | 1295.5 |
| B-1-4 | -691.7 | 1389.3 | -691.5 | 1389 | E-1-2 | -679.7 | 1381.4 | -645.9 | 1313.7 |
| B-1-5 | -712.4 | 1432.8 | -725.1 | 1458.3 | E-1-3 | -677.4 | 1372.9 | -651.8 | 1321.7 |
| B-1-6 | -712.4 | 1430.8 | -725.1 | 1456.3 | E-1-4 | -687 | 1390.1 | -663.1 | 1342.1 |
| C-0 | -673.9 | 1367.9 | -653.2 | 1326.5 | E-1-5 | -687.2 | 1392.3 | -663.1 | 1344.1 |
| C-1 | -687.2 | 1388.5 | -663 | 1340 | - | - | - | - | - |
