# Supplementary material for: Genetic Analysis and QTL Mapping of Fruit Peduncle Length in Cucumber (Cucumis sativus L.)
Source: PLoS One. 2016 Dec 9;11(12):e0167845. doi: 10.1371/journal.pone.0167845 (PMC5148027; doi:10.1371/journal.pone.0167845)
Supplement: S3 Table — U12, U22, U32 are the statistic of Uniformity test; nW 2 is the statistic of Smimov test; Dn is the statistic of Kolmogorov test. The critical of nW 2 is 0.461 at 0.05 level. * indicates the different significance at P <0.05 level. (DOCX) [file pone.0167845.s004.docx]

**S3 Table. Tests for goodness of fit of the preliminary selected inheritance model at the two sites.**

| Time | Model | Generations | *U*_1_^2^ | *U*_2_^2^ | *U*_3_^2^ | *D_n_* | *_n_W* ^2^ |
| --- | --- | --- | --- | --- | --- | --- | --- |
|  | D-1 | P1 | 1.363(0.2430)* | 1.949(0.1627)* | 1.128(0.2882)* | 0.3664(>0.05) | 0.1945(>0.05) |
|  |  | P2 | 0.060(0.8608) | 0.000(0.9965) | 0.931(0.3346)* | 0.1753(>0.05) | 0.0579(>0.05) |
|  |  | F1 | 1.244(0.2648)* | 1.397(0.2372)* | 0.167(0.6826) | 0.2757(>0.05) | 0.1453(>0.05) |
| 2014-Hainan |  | B1 | 1.633(0.2013)* | 1.581(0.2086)* | 0.006(0.9366) | 0.1331(>0.05) | 0.2425(>0.05) |
|  |  | B2 | 0.213(0.6446) | 0.395(0.5297) | 0.529(0.4669)* | 0.0979(>0.05) | 0.0895(>0.05) |
|  |  | F2 | 0.000(0.9897) | 0.002(0.9655) | 0.015(0.9023) | 0.0415(>0.05) | 0.0341(>0.05) |
|  | D-2 | P1 | 1.363(0.2429)* | 1.949(0.1627)* | 1.128(0.2881)* | 0.3665(>0.05) | 0.1926(>0.05) |
|  |  | P2 | 0.060(0.8067) | 0.000(0.9966) | 0.931(0.3346)* | 0.1753(>0.05) | 0.0579(>0.05) |
|  |  | F1 | 1.244(0.2647)* | 1.397(0.2372)* | 0.167(0.6826) | 0.2757(>0.05) | 0.1453(>0.05) |
|  |  | B1 | 1.633(0.2013)* | 1.580(0.2087)* | 0.006(0.9369) | 0.1331(>0.05) | 0.2425(>0.05) |
|  |  | B2 | 0.213(0.6446) | 0.395(0.5297) | 0.529(0.4669)* | 0.0979(>0.05) | 0.0895(>0.05) |
|  |  | F2 | 0.000(0.9897) | 0.002(0.9656) | 0.015(0.9022) | 0.0415(>0.05) | 0.0342(>0.05) |
|  | D-2 | P1 | 0.065(0.7989) | 0.009(0.9240) | 0.366(0.5451) | 0.1861(>0.05) | 0.0594(>0.05) |
|  |  | P2 | 0.122(0.7273) | 0.063(0.8014) | 0.119(0.7307) | 0.2078(>0.05) | 0.0586(>0.05) |
|  |  | F1 | 0.037(0.8475) | 0.225(0.6350) | 1.331(0.2486)* | 0.2376(>0.05) | 0.0570(>0.05) |
| 2015-Beijing |  | B1 | 2.826(0.0928)* | 2.632(0.1047)* | 0.000(0.9833) | 0.1071(>0.05) | 0.3150(>0.05) |
|  |  | B2 | 0.613(0.4335)* | 0.475(0.4905) | 0.076(0.7834) | 0.1443(>0.05) | 0.1872(>0.05) |
|  |  | F2 | 0.412(0.5210) | 0.661(0.4163)* | 0.585(0.4442)* | 0.0528(>0.05) | 0.0886(>0.05) |
|  | D-4 | P1 | 0.155(0.6938) | 0.008(0.9281) | 0.695(0.4045)* | 0.2029(>0.05) | 0.0681(>0.05) |
|  |  | P2 | 0.118(0.7310) | 0.036(0.8486) | 0.322(0.5704) | 0.2077(>0.05) | 0.0602(>0.05) |
|  |  | F1 | 0.071(0.7895) | 0.243(0.6221) | 0.879(0.3484)* | 0.2331(>0.05) | 0.0530(>0.05) |
|  |  | B1 | 2.682(0.1015)* | 2.500(0.1138)* | 0.000(0.9850) | 0.1052(>0.05) | 0.3008(>0.05) |
|  |  | B2 | 0.421(0.5162) | 0.238(0.6257) | 0.318(0.5731) | 0.1437(>0.05) | 0.1676(>0.05) |
|  |  | F2 | 0.405(0.5244) | 0.653(0.4190)* | 0.588(0.4432)* | 0.0526(>0.05) | 0.0879(>0.05) |

U12 , U22, U32 are the statistic of Uniformity test; nW 2 is the statistic of Smimov test; Dn is the statistic of Kolmogorov test. The critical of nW 2 is 0.461 at 0.05 level. * indicates the different significance at P <0.05 level
