# Supplementary material for: Genetic Analysis and QTL Mapping of Fruit Peduncle Length in Cucumber (Cucumis sativus L.)
Source: PLoS One. 2016 Dec 9;11(12):e0167845. doi: 10.1371/journal.pone.0167845 (PMC5148027; doi:10.1371/journal.pone.0167845)
Supplement: S4 Table — Loci with asterisks show segregation distortion. (DOCX) [file pone.0167845.s005.docx]

**S4 Table. Information of 78 SSR markers placed on the 1101×1694 linkage map.**

| # | Loci | LG(Chr) | cM | 9930 V2_Pos | Forward primer 5'-3' | Reverse primer 5'-3' |
| --- | --- | --- | --- | --- | --- | --- |
| 1 | SSR03462 | Chr1 | 0 | 2709412 | CAC gCg AgA ggA gAg gAT Ag | gAA ACA AAA TCT ggg gTC CA |
| 2 | SSR10018 | Chr1 | 8 | 4163912 | CTT TTg TTC TTg Tgg AAT gTg A | ATT Tgg ggA Tgg AgA ggT TC |
| 3 | CMBR40 | Chr1 | 9.5 | 4553412 | CgA CAA TCA Cgg gAg AgT TT | TTg TTg CAT CAA ACT AAC ACA ATC |
| 4 | CMBR97 | Chr1 | 9.5 | no hits | CgA CAA TCA Cgg gAg AgT TT | CAT ATT AgA CCC ATA TTT gTT gCA T |
| 5 | SSR01115 | Chr1 | 13.9 | 5233063 | ATT CCC AAT CCC AAA AAg gT | CTC CTC CTC CAA TgA gCA Ag |
| 6 | SSR16881 | Chr1 | 22 | 6858338 | CCC TCT CAA CAT TTT CCA CAA | CgA ggA gAC TTg ATg ggA Tg |
| 7 | SSR22637 | Chr1 | 22.4 | 6818593 | AAA AAT Cgg TTC AAC CCA AC | ACT TCC AAC CCA ACg ACT gT |
| 8 | SSR04992 | Chr1 | 24.3 | 7845023 | TTg ACC CAT AgA CCC ACA CA | AAC AAC CAA CAg gCC AAg Ag |
| 9 | SSR10134 * | Chr1 | 33.3 | 8146471 | CCA AAA CCA AAA gCA AAA TCC | AAA TTT gCC Agg AAC ACC Ag |
| 10 | SSR16055 | Chr1 | 39.9 | 13303381 | CAT CCT TTC gAC TTT gAT gC | CTg CAA ACg TgA AgA gAg CC |
| 11 | (280-282)STS | Chr1 | 40.1 | 13473574 | AAC ACT CCT gCT TTA ACA gCA TC | AAT gTA ATC gTC ATT CAg CAg TgT |
| 12 | 1CS6 | Chr1 | 40.3 | 13169701 | ggA ggA ggT ACA Tgg CTT Tg | TgA ATC TgA TCT TTC TAC CTT CAC A |
| 13 | SSR18530 | Chr1 | 58.6 | 18980946 | CCA CAg TCC CAC ACA CAA Ag | ggC gTT TTg TgA AgA CAg ATT |
| 14 | SSR03860 | Chr1 | 66.2 | 21159054 | gCC CTT ATT AAC CCA AgT TgC | AAT TgA ggg gCA CTT ATT gg |
| 15 | SSR14445 | Chr1 | 70 | 21848054 | TCC ATg gAA ATT gAA AAC CC | CgA TCC TTA TCg AAC AgC CT |
| 16 | SSR17922 | Chr1 | 83.9 | 24482692 | CAT TCT Agg TCA ATg AAT CgC A | gCA AAg TTg CCA CAT TgA Ag |
| 17 | SSR05748 | Chr2 | 0 | 89863 | TgT ggC CTg TgC TAA AAT gA | TTT ggA AAA gCT AAA gCC CA |
| 18 | SSR11097 | Chr2 | 3.131 | 605228 | TgA CAg AAg TCT ATg TgT TTC CAA | CCA AAT TCA AgA TCC ACT CCA |
| 19 | SSR06576 | Chr2 | 9.164 | 1746059 | TgA TCA Tgg gAA gAg AgA gAC A | TCA AgA AAT gTg ATg AAT ggA AA |
| 20 | SSR00204 | Chr2 | 18.788 | 4182584 | AAC CCT ATT TgC ACg CAT TC | gAg AAA CAg CTg gAA TTg gg |
| 21 | SSR13532 | Chr2 | 26.166 | 5647302 | AAA CCC AAg AAT TgT AAA CCC A | TgA TCC ATT TCT ATT CCT AAC ATT gA |
| 22 | SSR20268 | Chr2 | 54.844 | 11468334 | gCA ggA TAT ATT TCg AAA ACT gAA A | AAg TgA ATT gCT AAC AAg AAA Cg |
| 23 | SSR22203 | Chr2 | 66.428 | 13938767 | ggT gAg CAA ggg TTT TCT Tg | AAg gCg TTC CgA TgA TTT TT |
| 24 | SSR13818 | Chr2 | 70.179 | 14974226 | TTg TTA gTT CAT TTg Agg TgT CAA g | TCC ATA TTA ACT CTC TCA ggC TAA CA |
| 25 | SSR21502 * | Chr2 | 108.772 | 18100168 | Cgg gTC AAT TCC ATA TAT TAC AAA | AAA Agg gTA ATC gTT AgA AAC AgA A |
| 26 | SSR21055 * | Chr3 | 0 | 1904226 | CAT CCT CAT CCA CCC AAT CT | TCC AAA CAA gTC TgA CAA ggA A |
| 27 | SSR28074 | Chr3 | 15.57 | 2555132 | CCC TAg ATT ggC TgA TCC Tg | TCC CgA AAT CTg TTC TAC Cg |
| 28 | SSR01573 * | Chr3 | 40.701 | 8399827 | CgT TAg gCC AAA CAA AAT TgA | TgC AAA CgT TTC TCT Agg CA |
| 29 | SSR18861 * | Chr3 | 57.986 | 11072331 | TCC CAC CAT CTT CTC gTA gg | TTC AAA gCT gTg gCT gTg TT |
| 30 | SSR14649 * | Chr3 | 94.628 | 22876104 | AAA ACA CCC AAA TTT TAA gCg A | CAC CAT TAA TTA ACg ATC AAC CA |
| 31 | SSR10357 | Chr3 | 102.578 | 24792526 | AAg AAg TAT gCg Agg TAA ggg A | gCC AAg AAg TAA gAA gTg gTT gA |
| 32 | SSR11699 | Chr3 | 109.453 | 26707596 | CAC gTA TCA AAT gTA TAT gAA ggg | TgC AAA ACg TgA AAA ACA gg |
| 33 | SSR16667 | Chr3 | 113.133 | 27747223 | TTT gCA gAg ggC AAA gAA Ag | TCg gTg gAg ggA TTC ATA AC |
| 34 | SSR18719 | Chr4 | 0 | 22116 | TTT Agg TCC gAC ATC TTC ATT T | CgA TgA TTC TCA CAT TTT Tgg A |
| 35 | SSR23549 | Chr4 | 10.108 | 1063659 | TCA CCC CCA CTT TAC TCC TC | AgT CAA TCA gTC AgC gCC TT |
| 36 | SSR22231 | Chr4 | 12.883 | 1654406 | CAT gTg TgA CgC TTC TTT ATT TTT | Tgg TgC TCC CTT TCT ACA CA |
| 37 | SSR07431 | Chr4 | 32 | 3893349 | AAA TgA TAA ATg CAA AAg CAC g | TgA AAg Agg Agg CAg TTT CC |
| 38 | SSR22706 | Chr4 | 38.708 | 5070734 | CAT AAg CCT TTC AAg CTg gg | CTg Agg TCT CCT gAT ggA gg |
| 39 | SSR14617 | Chr4 | 76.683 | 11650004 | CAA CTC Cgg TTC AAA AgT TCA | TgT CTT CAA TgC CCT TTC Ag |
| 40 | SSR15737 | Chr4 | 83.481 | 13104405 | TCC CAC AAC TTC AAT gAg CA | TgT Cgg TTC ACT gTC AAT CTT |
| 41 | SSR00185 | Chr4 | 98.624 | 15166131 | AAg TgC CAT ggT gAA Tgg TT | TgC ATg ggA TAC CTC AAC AA |
| 42 | SSR26165 | Chr4 | 99.48 | 15188605 | TCT TTT gTT ggT gAA AAT TgA AA | CCT TCT CAT gTg TAT TgT CTT TTg |
| 43 | SSR17406 | Chr4 | 120.663 | 21106727 | gAg CCA TCC ATC AgA gAg AgA | ACC CAC AAg CTT CAg Agg TC |
| 44 | SSR11969 | Chr5 | 0 | 5974050 | ggg TCA TAC CCA AAA ggg Ag | TgC TTT AgC CgA CAA CTC AA |
| 45 | SSR00023 | Chr5 | 5.274 | 6627302 | gCA Cgg ATT CTg ggT TTA gA | TCA TCC TTC ggA ATC gTA CA |
| 46 | SSR06447 | Chr5 | 15.813 | 8580058 | AAg TAT gAC gAC ACC CTT Cg | CgC AAA ACC gAA Agg TAC ATA |
| 47 | SSR19178 | Chr5 | 40.2 | 13662472 | CCg TTC CTA gAA TAg CgT gT | CCC ACg CCT TCT ATT CTA CA |
| 48 | SSR07531 * | Chr5 | 59.382 | 17208433 | gAg TCC gTT ggC TTA TTT CAA | TCC CTC TTT gTC ggT CCA TA |
| 49 | SSR16203 * | Chr5 | 78.432 | 20451369 | TCg Agg TAA ATC AAA ACC gA | ATg TgT CAA ACC CAC CCA TT |
| 50 | SSR23265 | Chr5 | 96.233 | 23692227 | CCT CCC TCC ATT TTC CAC TA | TgA gCC AgT Tgg ggT TTT Ag |
| 51 | SSR02459 | Chr5 | 113.3 | 27816197 | TCg gAA gAT ggg TTA TTT gg | TgA CCC CTC ACA TTC TCT CC |
| 52 | SSR02021 | Chr6 | 0 | 997081 | TAA ACA Tgg CTT CCT CCT CC | CTC TCT TTT CTC ACA CCC ACA g |
| 53 | SSR02763 | Chr6 | 42.133 | 6444729 | ggg ACC TCT gAg CTT TTT CC | AAA ACg CAA ATg ggT ATT Cg |
| 54 | SSR21186 | Chr6 | 55.885 | 9198353 | TTT gAg CAA CAC TTC gCA AC | gCA TgT TTT CAT gTC ATT ggA |
| 55 | SSR13884 | Chr6 | 62.241 | 11069339 | ggA TAA TCC TgA TTC CTg Tgg | TgT gCA ACT gAA AAC gAA gC |
| 56 | SSR15067 | Chr6 | 67.084 | 13578213 | AAC CAC TCC CAC TTg CCT AA | AAA AAT ATT CAA ACC CAA TTT TTg A |
| 57 | SSR12834 | Chr6 | 81.932 | 18735018 | CAA AgA ggC CAA TTC TCC Ag | Tgg Tgg AAg ATC AAA gAg gC |
| 58 | SSR15955 | Chr6 | 83.339 | 18995075 | TTT gAg CCT TgA ggC AAA gT | gCA ATT CAA CgT AAT ggg CT |
| 59 | SSR14859 | Chr6 | 92.345 | 20591185 | CAA ACC TAg CCT TAA CCT TTg g | AAA CCT TTT TgA CgT TgA Agg A |
| 60 | SSR11985 | Chr6 | 93.538 | 20659656 | gCT gCA TTT CAT TTA ACg CTT | Tgg TCC ATC CTC ACC AAT TT |
| 61 | SSR19165 | Chr6 | 97.152 | 21344610 | AAT CCA CgT Tgg TTg TCg TT | gAA ggg CCA AAA ATg TTT CA |
| 62 | SSRFPL-1 | Chr6 | 106.214 | 22690272 | GCGTGTGATGGAACTAGAG | ACGGCTTCTATTACATCTACC |
| 63 | UW021226 | Chr6 | 111.575 | 23873711 | TGTGCAGTAGGCCATGAAAG | TTCTCCTCTGTTCCCCATTG |
| 64 | SSR15516 | Chr6 | 114.913 | 24469033 | TgA ggg TTT AAA AgA AAA Agg Tg | gCC AAT TCC CCA ATT CTT AAT |
| 65 | SSRFpl-32 | Chr6 | 116.375 | 25277182 | GGTTGTTCGTGGTTGAATG | AGCCAGGACTTGGATTACT |
| 66 | SSR13611 | Chr6 | 118.333 | 25498366 | ACg ggA ACA CTC ATg TCT CA | TTT CgT TgT TTA gTA gAC gTT AAA AgA |
| 67 | SSR16882 | Chr6 | 125.94 | 27767471 | CAC CTC AAC TCC TCC ATT CAA | Tgg Agg TCA TTg AgA CTT gCT |
| 68 | SSR17370 | Chr6 | 126.477 | 28561745 | Cgg Cgg Agg TTT gAT AAg TA | TTT TTC TgA ACT CCC AAg ggT |
| 69 | SSR00193 | Chr6 | 130.08 | 28813523 | gCC AAT CCA ATg gAA CAA gT | TTg TAA ACC AAA ACC TTA CCC C |
| 70 | SSR03357 | Chr6 | 130.823 | 28813523 | AAA Agg gCA AgT CAA AAC CC | ggg gAg gAA gAg AgA CCC TT |
| 71 | SSR02821 * | Chr6 | 133.942 | no hits | AAA ATg ggA ACg AAg gAC AA | TCC CTC CCA CCC TAT TTT TC |
| 72 | SSR07473 * | Chr7 | 0 | 6781166 | CgA gTg Tgg ACA TTT TAg Agg g | TTg ACT TTC CTC ATT CTT TTA ATg C |
| 73 | SSR00048 | Chr7 | 21.116 | 12334249 | CCg ATC gTT gCT Agg AgA AC | CTC CCg AAA CCT AgA AAC CC |
| 74 | SSR00215 | Chr7 | 21.433 | 11723991 | ggA gCC CTA gTA ggA AAC Cg | ggA CCA CgT gAA AgA TTC AgA |
| 75 | SSR14606 | Chr7 | 26.552 | 13987263 | TCT CAT ggA TCA TTA TTg ggC | CCC AAC AAA AgA AAT gCT CA |
| 76 | SSR22593 | Chr7 | 30.614 | 14743975 | CgT TgC ATg gAT AAA gAA ACC | ATT ATg TTC CAg CTC CAC gg |
| 77 | CSWCT11 | Chr7 | 36.322 | 15794858 | ATA ggC AAT ATg gCT TCT | CAC TTC AAT ggA gTT TCg |
| 78 | SSR20122 | Chr7 | 46.916 | 19152348 | TTC CgT gTg TTT AgT TAg TTT ATT TCA | gAA CTC AAC AAA ATg gAg TTC AgA |

*Loci with asterisks show segregation distortion
